# Dexpanthenol Promotes Cell Growth by Preventing Cell Senescence and Apoptosis in Cultured Human Hair Follicle Cells

**DOI:** 10.3390/cimb43030097

**Published:** 2021-09-28

**Authors:** Jae Young Shin, Jaeyoon Kim, Yun-Ho Choi, Nae-Gyu Kang, Sanghwa Lee

**Affiliations:** LG Household & Health Care (LG H&H) R&D Center 70, Magokjoongang 10-ro, Gangseo-gu, Seoul 07795, Korea; sjy2811@lghnh.com (J.Y.S.); kjy5281@lghnh.com (J.K.); youknow@lghnh.com (Y.-H.C.)

**Keywords:** D-panthenol, dermal papilla, outer root sheath, anti-hair loss, anagen, follicle aging

## Abstract

Dexpanthenol (D-panthenol) is a precursor of vitamin B5 (pantothenic acid) and is widely used for dietary supplements and topical applications. D-panthenol has long been used in hair care products for the purpose of anti-hair loss, its effects and the underlying mechanisms, however, were barely reported. In this study, the effects of D-panthenol on human hair follicle cells, including dermal papilla cells (hDPCs) and outer root sheath cells (hORSCs), were investigated. D-panthenol enhanced the cell viability, increasing the cellular proliferation marker Ki67 in cultured hDPCs. The markers for apoptosis (Caspase3/9) and cell senescence (p21/p16), reported to be expressed in aged or resting phase follicles, were significantly reduced by D-panthenol. Anagen-inducing factors (ALP; β-catenin; versican), which trigger or elongate the anagen phase, were stimulated by D-panthenol. On the other hand, D-panthenol reduced TGF-β1 expressions in both mRNA and protein levels. The expression of VEGF, which is important for peripheral blood vessel activation; was up-regulated by D-panthenol treatment. In cultured hORSCs, cell proliferation and viability were enhanced, while the mRNA expression of cell senescence markers (p21/p16) was significantly down-regulated. The expressions of both VEGF and its receptor (VEGFR) were up-regulated by D-panthenol. In conclusion, our data suggest that the hair growth stimulating activity of D-panthenol was exerted by increasing the cell viability, suppressing the apoptotic markers, and elongating the anagen phase in hair follicles.

## 1. Introduction

Anagen is a specific period occupying the majority of the hair cycle. In anagen, hair follicular cells go through vigorous cell proliferation and differentiation to make the hair shaft [1]. During catagen and telogen, cells in the lower part of the hair follicle go through apoptosis, which leads to hair follicle shrinkage and hair shaft loss, respectively [2]. Given the clinical importance of the anagen-catagen transformation in human hair growth disorders, the abnormal termination of the anagen phase triggers gradual hair thinning [3]. In this context, it has long been regarded that extension of the anagen phase is a main strategy of anti-hair loss treatment.

The dermal papilla and outer root sheath are the special compartments of the hair follicle, playing important roles in hair follicle morphogenesis and regeneration. Previous studies suggested that the anti-apoptotic and proliferative potencies of dermal papilla cells are the first clue for anti-hair loss property [4]. In particular, alkaline phosphatase (ALP) activity in dermal papilla has been regarded as an important factor for the progression of the hair follicle cycle inducing hair follicle neogenesis and telogen to anagen transition [5]. Growth factors from the dermal papilla, including insulin-like growth factor-1 (IGF-1), vascular endothelial growth factor (VEGF) and keratinocyte growth factor (KGF) stimulate matrix keratinocytes to proliferate and differentiate into the hair shaft during anagen [6,7,8]. The transition of hair cycles is also mainly controlled by the dermal papilla. Secreted proteins, such as transforming growth factor (TGF) β1, TGF β2, and dickkopf1 (DKK-1) are known to induce the transition from anagen to telogen [9,10,11]. On the other hand, the outer root sheath (ORS), which takes a mold part of the hair follicle structure, plays a connective role between bulge stem cells and bulb dermal cells (dermal papilla and dermal sheath cells) in the anagen phase [12]. When the telogen to anagen transition signaling begins, bulge regional outer root sheath cells begin to proliferate and to differentiate downward to activate the dermal papilla [13]. Once the ORS cells escape from quiescent/conserved status, cellular proliferative and anti-apoptotic characteristics are more important than stem cell characteristics for maintaining the anagen phase. While several markers, such as SOX9, CD34, and Keratin 15 in the bulge region show stem cell lineage maintenance functions, cellular activation markers, such as Ki67 and BCL2, in the lower part of ORS are considered as proliferative and anagen-elongating factors [12,14]. This differentiated compartment of the ORS could interact only with the basal part of the hair follicle, the dermal papilla.

D-panthenol, the alcohol form of pantothenic acid (Vitamin B5), is a well-known cosmetic and pharmaceutical ingredient used for anti-inflammatory, skin regenerating, and stratum corneum hydrating effects [15,16,17]. Because of these skin improving properties, D-panthenol is widely used to relieve atopic dermatitis, nappy rash symptoms, and sunburns [18,19]. D-panthenol was also reported to facilitate wound repair by up-regulating wound-healing associated molecules, such as IL-6, IL-1β, CYP1B1, CXCL1, CCL18 and KAP4-2 [20,21].

In addition, D-panthenol has long been used for hair and scalp care products, especially for the purpose of anti-hair loss. Several clinical studies reported that the oral administration of D-panthenol improved female pattern hair loss as well as male androgenetic alopecia [22,23]. Its effects on hair follicle cells and underlying mechanisms, however, were barely reported.

In this study, we investigated the in vitro hair growth-promoting properties of D-panthenol in cultured hDPCs and hORSCs. The effects of D-panthenol on the cell growth and the expression of apoptosis/cell senescence related genes were measured to elucidate cellular proliferative properties. The expression levels of anagen and telogen markers were assessed to examine the effect of D-panthenol on hair follicle cycle modulation. The effect of D-panthenol on the expression of several growth factors and receptors, including TGF-β1, VEGF and VEGFR, were also evaluated.

## 2. Materials and Methods

### 2.1. Culture of Human Dermal Papilla Cells (hDPCs) and Outer Root Sheath Cells (hORSCs)

hDPCs were purchased from Promocell (Heidelberg, Germany) and hORSCs were purchased from ScienCell research laboratories (Carlsbad, CA, USA). hDPCs were cultured in a basal medium supplemented with Supplement Mix, which contains 4% fetal calf serum, 0.4% bovine pituitary extract, 1 ng/mL of basic fibroblast growth factor, and 5 μg/mL of insulin. hORSCs were cultured in a mesenchymal stem cell medium containing 5% FBS, 1% penicillin streptomycin and 1% MSCGS (Mesenchymal Stem Cell Growth Supplement) which is provided by the manufacturer. Cells were maintained in a humidified incubator at 37 °C with 5% CO_2_. hDPCs under the passage number 5 were used for experiments.

### 2.2. Cell Viability Assay

D-panthenol was purchased from xinfa pharmaceutical (Shandong, China) and the stock solution was prepared by dissolving in DMSO. The effects of D-panthenol on cell viability were examined using CCK-8 assay (Dojindo, Rockville, MD, USA), following the manufacturer’s protocols. Briefly, hDPCs (3 × 10^3^ cells/well) and hORSCs (3 × 10^3^ cells/well) were seeded in 96-well plates and cultured for 24 h. Triplicate cultures of hDPCs and hORSCs were treated with various concentrations of D-panthenol and cultured for another 24 h. The generation of NADH and NADPH was determined by CCK-8 assay which indicates the cell viability. The absorbance at 450 nm was measured using an Epoch micro plate spectrophotometer (BioTek, Winooski, VT, USA). Minoxidil was purchased from Sigma Aldrich (St. Louis, MO, USA) and the stock solution was prepared by dissolving in DMSO.

### 2.3. mRNA Analysis

hDPCs (1 × 10^6^ cells/well) and hORSCs (1 × 10^6^ cells/well) were seeded in 6-well plates and cultured for 24 h. Then D-panthenol was treated at appropriate concentrations for 24 h. Total RNA was isolated using an RNA isolation kit (Qiagen, RNeasy mini kit), according to the manufacturer’s guide. After RNA isolation, cDNA was synthesized by reverse transcription using an eCube cDNA synthesis kit (philekorea, Korea) with a PCR thermocycler (R&D systems, Minneapolis, MN, USA), according to the manufacturer’s protocol. cDNA obtained from control cells and D-panthenol treated cells were subjected to real-time PCR analysis. TaqMan probes used in this study were as follows: GAPDH assay id 4352934E; Caspase3 assay id Hs00234387_m1; Caspase 9 assay id Hs00962278_m1; CDKN1A assay id Hs00355782_m1; CDKN2A assay id Hs00923894_m1; ALP assay id Hs01029144_m1; Versican assay id Hs00171642_m1; TGF-β1 assay id Hs00998133_m1; Bcl2 assay id Hs00608023_m1; Bax assay id Hs00180269_m1; VEGFA assay id Hs00900055_m1; VEGFR (KDR) assay id Hs00911700_m1. TaqMan One-Step RT-PCR Master Mix Reagents (Life Technologies, Carlsbad, CA, USA) was used. The PCR reactions were performed on an ABI7500 Real Time PCR system following the manufacturer’s protocol. The resulting data were analyzed with ABI software (delta-delta Ct method).

### 2.4. Western Blot Analysis

hDPCs (1 × 10^6^ cells/dish) were seeded in 100 mm culture dishes and cultured for 24 h. D-panthenol was treated at appropriate concentrations for 24 h. Cells were then washed with ice-cold PBS and lysed on ice in an M-PER buffer (Thermo Fisher Scientific, Waltham, MA, USA) supplemented with a Complete™ protease inhibitor cocktail and phosphatase inhibitor (Roche, Indianapolis, IN, USA). 40 μg of protein was analyzed by Western blotting with appropriate antibodies to evaluate protein expression; β-catenin (1000:1 dilution, Santa Cruz, CA, USA), CDKN2A/p16INK4a (1000:1 dilution, Abcam, Cambridge, UK), p21 (1000:1 dilution, cell signaling technology, Danvers, MA, USA), caspase3 (1000:1 dilution, cell signaling technology, Danvers, MA, USA), GAPDH (2000:1 dilution, Santa Cruz, CA, USA). Western blot was analyzed by a chemiluminescence detector (iBright FL1500, Thermo Fisher Scientific, Waltham, MA, USA).

### 2.5. Immunocytochemistry

hDPCs (5 × 10^4^ cells/well) were seeded in 24-well plates. After 24 h, cells were treated with appropriate concentrations of D-panthenol for 24 h. After an ice cold PBS wash, hDPCs were fixed with 4% paraformaldehyde at room temperature for 10 min. Cells were then permeabilized with PBS containing 0.1% triton X-100 and blocked with PBS containing 5% FBS and 1% BSA. After consecutive incubation with the primary antibodies (200:1 dilution, Abcam, Cambridge, UK) at 4 °C for 12 h and the alexa 488 nm or alexa 594 nm conjugated secondary antibodies (1000:1 dilution, Thermo Fisher Scientific, Waltham, MA, USA) at room temperature for 1 h, nucleus were stained with DAPI (2000:1 dilution, Thermo Fisher Scientific, Waltham, MA, USA) in the dark for 10 min. High resolution fluorescence images were taken using the EVOS^TM^ FL Auto2 Imaging System (Thermo Fisher Scientific, Waltham, MA, USA).

### 2.6. Alkaline Phosphatase (ALP) Staining and Quantification

hDPCs (5 × 10^3^ cells/well) were plated in 96-well plates and cultured for 24 h. Cells were treated with D-panthenol for 24 h, and then fixed in 4% paraformaldehyde for 10 min and washed with PBS. For efficient staining, cells were permeabilized with PBS containing 0.1% triton X-100. After permeabilization, ALP was stained with Vector^®^Blue kit. Vector blue reagents 1, 2, and 3 were mixed in 200 mM of Tris-HCl (pH 8.5) solution. 100 μL of mixed ALP solution was treated to each well and incubated for 30 min in the dark. After treatment, the whole area of each well was measured by the EVOS^TM^ FL Auto2 Imaging System (Thermo Fisher Scientific, Waltham, MA, USA). To quantify the number of ALP positive cells and intensity, fluorescence images were obtained under the same conditions using 561 nm/594 nm (Excitation/Emission) wavelength which react with ALP staining. Celleste image analysis software was used for quantification (Thermo Fisher Scientific, Waltham, MA, USA).

### 2.7. Statistical Analysis

All experimental data were presented as the mean ± standard deviation (S.D.) of at least 3 independent experiments, otherwise indicated. Experimental results were analyzed using the SigmaPlot (Systat Software Inc., San Jose, CA, USA). The statistical significance of the difference was determined using Student’s *t*-test. The value of *p* < 0.05 was considered statistically significant.

## 3. Results

### 3.1. D-Panthenol Stimulated the Growth of Cultured hDPCs

**Figure 1:**
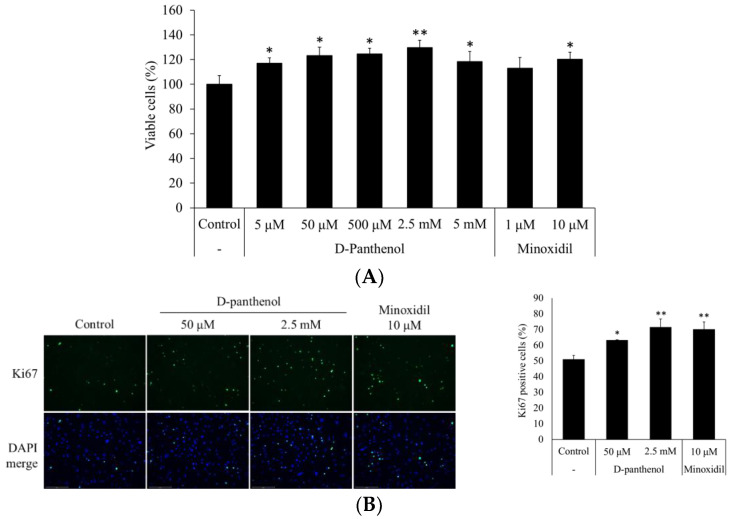
D-panthenol stimulated the growth of cultured human dermal papilla cells (hDPCs). (**A**) The growth of hDPCs was assessed by CCK-8 assay after 24 h treatment of D-panthenol. (**B**) Ki67, a cell proliferation marker was immunostained after 24 h treatment of D-panthenol and the proportions of Ki67 positive cells were calculated (×200 magnification). Ki67 positive cells were marked by green fluorescence and total cells were marked by DAPI. * *p* < 0.05, ** *p* < 0.01 versus control group. Data are expressed as mean ± SD.

To evaluate the cell proliferative effect of D-panthenol, hDPCs were treated with 5 μM to 5 mM of D-panthenol. The viability of hDPCs was measured by cell counting kit 8 (CCK8) assay after 24 h treatment. Minoxidil was used as a positive control. D-panthenol stimulated the growth of hDPCs in a concentration-dependent manner (Figure 1A). Maximal growth increment was approximately 30% at a 2.5 mM concentration. Also, fluorescent staining results showed that Ki67 positive cells were increased by the D-panthenol treatment (Figure 1B).

### 3.2. D-Panthenol Reduced Apoptotic and Senescence Markers in Cultured hDPCs

**Figure 2:**
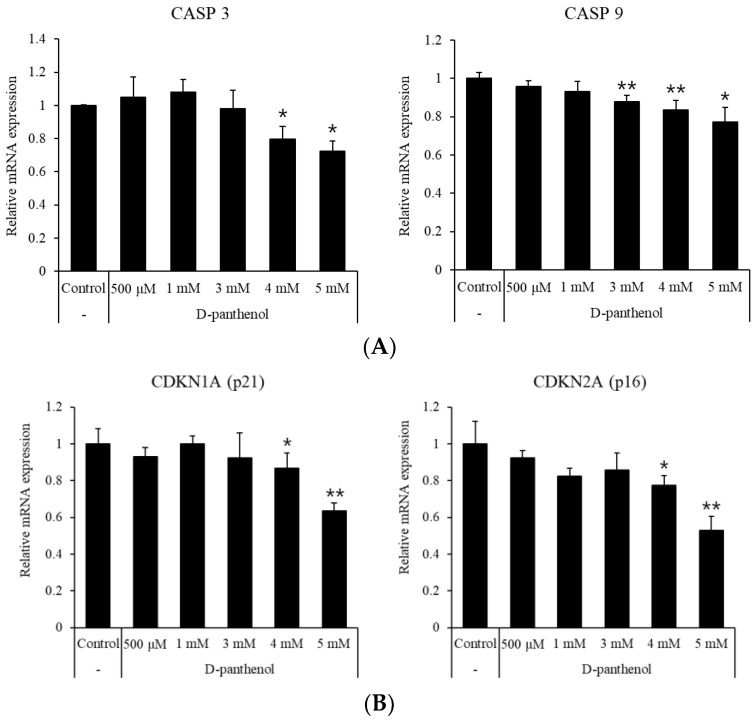

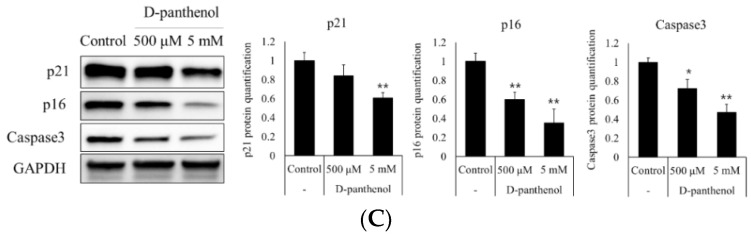
D-panthenol reduced apoptosis and senescence markers in cultured hDPCs. (**A**) mRNA expression levels of caspase 3, caspase 9 in hDPCs after 24 h treatment of D-panthenol. (**B**) mRNA expression levels of senescence markers (p21, p16) after 24 h treatment of D-panthenol. (**C**) Protein levels of p21, p16 and caspase 3 were analyzed by Western blot and quantitated. * *p* < 0.05, ** *p* < 0.01 versus control group. Data are expressed as mean ± SD.

Pantothenic acid was previously reported to prevent the apoptotic process induced by oxidative stress in several mammalian cell types [24]. Caspase 3 and caspase 9, which lead to cell apoptosis, are regarded as anagen termination markers [25]. During the catagen and telogen phase of the hair follicle, the miniaturization of the dermal papilla is committed by several molecules. The apoptosis signaling triggered by caspase 9 and ended by caspase 3 results in mitochondrial dysfunction [26]. As shown in Figure 1A, D-panthenol treatment increased the cell growth which is represented by mitochondrial function (CCK8). To evaluate whether up-regulated mitochondrial function is associated with apoptosis and senescence of hDPCs, the mRNA expressions of apoptosis—and senescence-related genes were measured after the D-panthenol treatment. The mRNA expressions of apoptosis markers, caspase 3 and caspase 9, were significantly reduced by 20% compared to the control (Figure 2A). Along with the anti-apoptotic effects, cell senescence markers, p21 and p16, were also significantly reduced by D-panthenol in concentration-dependent manners. Relative mRNA expressions of p21 and p16 were significantly reduced by 60% and 50%, respectively (Figure 2B). In addition, protein expression levels of p21, p16 and caspase 3 were significantly reduced by 60%, 40% and 50%, respectively (Figure 2C). These results indicate that D-panthenol could prevent catagen/telogen entry by preventing the cell senescence and apoptosis in dermal papilla.

### 3.3. D-Panthenol Significantly Increased the Anagen Markers in Cultured hDPCs

**Figure 3:**
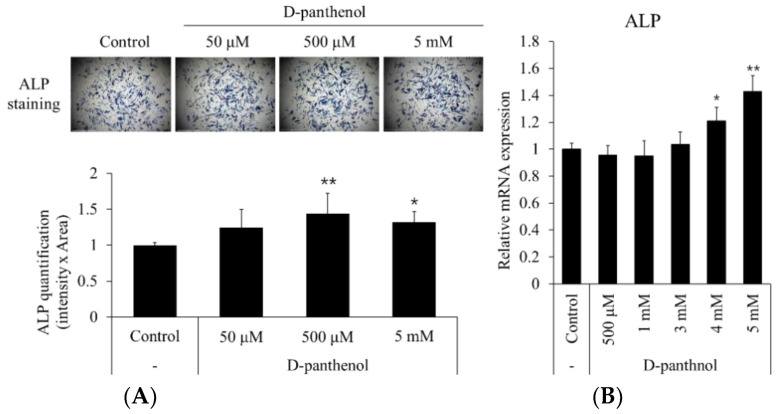

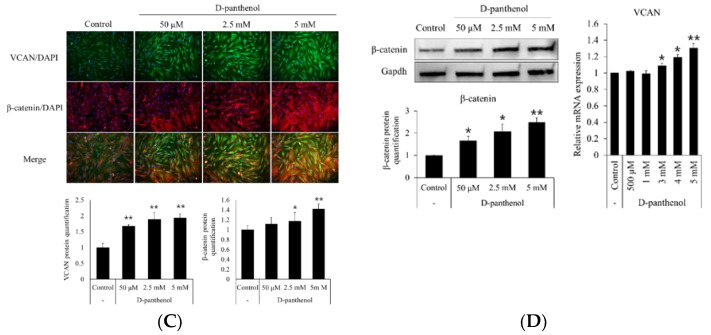
Anagen markers of hDPCs were significantly increased by D-panthenol treatment. (**A**) Alkaline phosphatase staining (×40 magnification) and (**B**) mRNA expression of ALP after 24 h treatment of D-panthenol in cultured hDPCs. (**C**) Versican and β-catenin expressions were double stained by immunocytochemistry after 24 h treatment of D-panthenol (×200 magnification). (**D**) Western blot analysis of β-catenin and RT-PCR analysis of versican mRNA were also performed. * *p* < 0.05, ** *p* < 0.01 versus control group. Data are expressed as mean ± SD.

To elucidate the correlation between D-panthenol-induced hDPC growth and hair cycle modulation (anagen and catagen), hDPC specific anagen markers, such as alkaline phosphatase (ALP) and β-catenin, were measured after the D-panthenol treatment. After 24 h treatment of 50 μM to 5 mM of D-panthenol, the number and intensity of ALP positive cells were significantly increased by 40% (Figure 3A). The mRNA expression level of ALP was also significantly up-regulated by 40% in 5 mM D-panthenol treated groups (Figure 3B). Alkaline phosphatase activity is considered as an inherent marker for hair growth promotion in dermal papilla [27]. Furthermore, ALP activity was reported to be regulated by Wnt/β-catenin pathway activation [28]. Histochemical studies revealed that both ALP and β-catenin were highly expressed in the early to mid-anagen phase of the hair follicle [27,29]. As shown in Figure 3C, immunocytochemical staining revealed that D-panthenol concentration dependently up-regulated the protein expressions of β-catenin and versican. Western blot and RT-PCR analyses confirmed the increased β-catenin protein and versican mRNA levels, respectively (Figure 3C).

### 3.4. D-Panthenol Reduced TGF-β1 Expression in Cultured hDPCs

**Figure 4:**
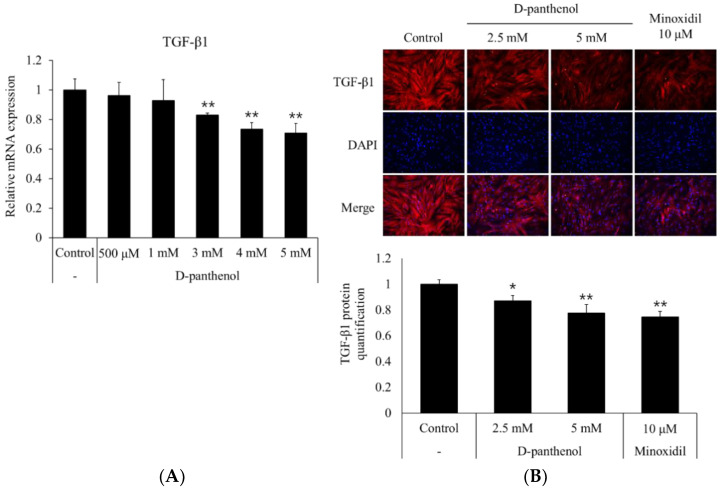
D-panthenol reduced TGF-β1 expression in cultured hDPCs. Treatment of D-panthenol for 24 h decreased the (**A**) mRNA and (**B**) protein expressions of TGF-β1 in cultured hDPCs (×200 magnification). * *p* < 0.05, ** *p* < 0.01 versus control group. Data are expressed as mean ± SD.

TGF-β1 was reported to stimulate the hair follicle to enter the catagen phase [30]. In androgenetic alopecia, precocious, repeated stimulation of the dermal papilla by androgens inhibits the growth of adjacent epithelial cells which leads to the progression of hormonal baldness [31]. To evaluate whether D-panthenol possibly inhibits the catagen entry of the dermal papilla, the effects of D-panthenol on the mRNA and protein expression levels of TGF-β1 were assessed. As shown in Figure 4A, 5 mM D-panthenol significantly decreased the mRNA expression of TGF-β1 in cultured hDPCs by 30% compared to the control. Immunocytochemistry results showed that D-panthenol also decreased the TGF-β1 protein level in a concentration-dependent manner (Figure 4B).

### 3.5. D-Panthenol Stimulated VEGF Expression in Cultured hDPCs

**Figure 5:**
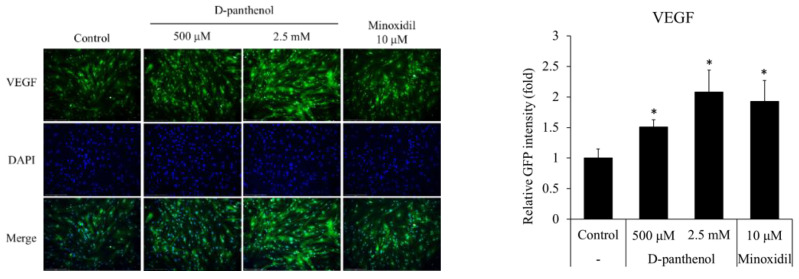
D-panthenol stimulated VEGF expression in cultured hDPCs. The protein level of VEGF in cultured hDPCs was evaluated by immunocytochemistry after 24 h treatment of D-panthenol (×200 magnification). * *p* < 0.05 versus control group. Data are expressed as mean ± SD.

In the hair follicle, VEGF plays a critical role for hair growth promotion, especially in anagen phase elongation [7,32,33]. VEGF from dermal papilla attracts adjacent blood vessel to supply vascular-derived growth factors to the hair follicles. VEGF also stimulates adjacent cells, such as outer root sheath cells, matrix progenitor cells, sebaceous glands, sweat glands, and epidermis to make the hair follicle fully matured and the hair shaft differentiated [34]. As shown in Figure 5, the protein level of VEGF was up-regulated by the D-panthenol treatment.

### 3.6. D-Panthenol Stimulated the Growth of Cultured hORSCs and Reduced the Senescence Markers

**Figure 6:**
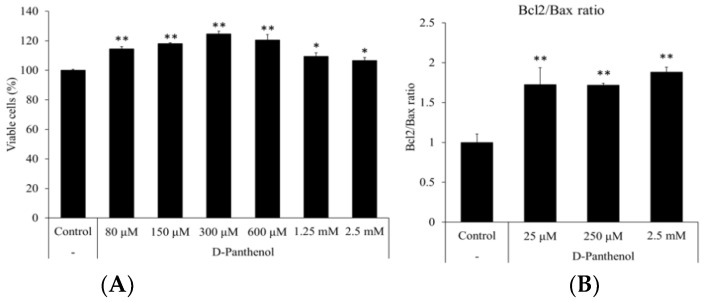

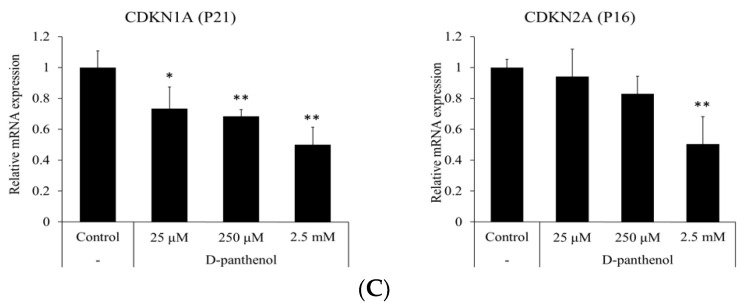
D-panthenol stimulated the growth of human outer root sheath cells (hORSCs). (**A**) The growth of hORSCs was assessed after 24 h treatment of D-panthenol. (**B**) Relative mRNA expression ratios of Bcl2 to Bax were calculated after 24 h treatment of D-panthenol. (**C**) mRNA expression of senescence markers (p21 and p16) in cultured hORSCs after 24 h treatment of D-panthenol. * *p* < 0.05, ** *p* < 0.01 versus control group. Data are expressed as mean ± SD.

The hORSCs are keratinocytes which originated from epidermal bulge stem cells and comprise the outermost part of the hair follicle. Hair follicle hORSCs go through repeating proliferation and apoptosis during hair follicle elongation and regression cycles, respectively [35]. During late telogen and early anagen, bulge stem cells migrate along the ORS to reach the hair bulb [36]. Because the proliferative capacity of hORSCs determines the anagen, catagen or telogen phase of the hair follicle structure, the effects of D-panthenol on the cell viability were investigated in several parameters [37]. The treatment of D-panthenol significantly stimulated the growth of cultured hORSCs in a concentration-dependent manner (Figure 6A). NADH and NADPH generation, presented as cell viability, were increased by 15% and 20% in the presence of 0.15 mM and 0.3 mM of D-panthenol, respectively. D-panthenol significantly increased the Bcl2/Bax ratio to 1.71 and 1.88 folds, suggesting a possible anti-apoptotic effect of D-panthenol in cultured hORSCs (Figure 6B). Furthermore, mRNA expressions of p21 and p16 were significantly reduced by 50% in 2.5 mM of D-panthenol treatment, indicating that cell senescence could be prevented by D-panthenol (Figure 6C).

### 3.7. D-Panthenol Induced the Expression of VEGF and VEGFR in Cultured hORSCs

**Figure 7:**
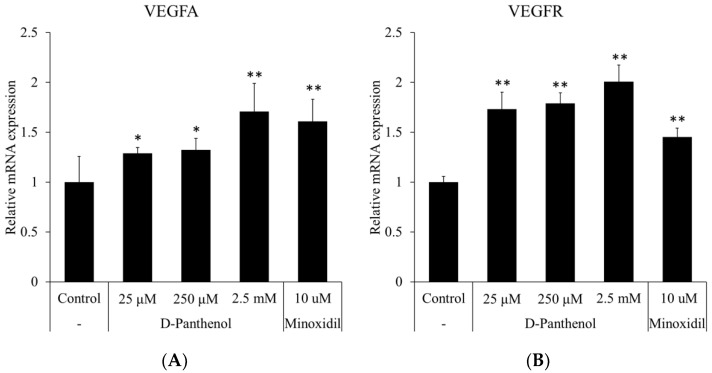
The mRNA expression levels of VEGF and VEGFR were up-regulated by D-panthenol treatment in cultured hORSCs. The mRNA expression of (**A**) VEGFA and (**B**) VEGFR (KDR) was evaluated by RT-PCR after 24 h treatment of D-panthenol. * *p* < 0.05, ** *p* < 0.01 versus control group. Data are expressed as mean ± SD.

Several reports demonstrated that VEGF mediated the proliferation of hORSCs through the ERK pathway [12]. To clarify the underlying mechanism for the proliferative effect of D-panthenol on hORSCs, changes in mRNA levels of several growth factors were evaluated. Of note, VEGFA expression was prominently increased by 70%, comparable to that of the minoxidil treatment, a positive control (Figure 7A). The expression of VEGF receptor 2 (KDR) was also significantly increased by D-panthenol in a concentration-dependent manner (Figure 7B). Our data indicate that D-panthenol induced the proliferation of cultured hORSCs through VEGF signaling.

## 4. Discussion

In the present study, we investigated the in vitro anti-hair loss effects of D-panthenol, focusing on the modulation of the hair follicle cycle in cultured human dermal papilla and outer root sheath cells. D-panthenol is widely used as a skin pharmaceutical and cosmeceutical ingredient. Most of the research on the pharmacological action of D-panthenol is mainly focused on skin hydration, anti-irritation and wound healing. Little was done in the field of anti-hair loss research, especially concerning the cell-based mechanisms, despite its wide use in hair and scalp care products.

We have found that D-panthenol enhanced the growth of hDPCs and hORSCs (Figure 1A and Figure 6D), inducing the expression of cell proliferation marker (Ki67, Figure 1B) in cultured hDPCs. Cellular growth in hair follicles has been reported as a significant characteristic of the anagen phase [38,39,40,41]. The dermal papilla goes through miniaturization when hair follicles proceed to catagen and telogen [42,43], after which it recovers its original size in the course of anagen initiation. The recovering growth force of the dermal papilla could grant anagen persistency against several insults, such as DHT, TGF- β, and oxidative stress. Unlike the dermal papilla, the outer root sheath establishes the outer structure of the hair follicle from the upper bulge to lower bulb through the most vigorous proliferation during the anagen phase [44]. The outer root sheath, on the other hand, does not go through miniaturization, but instead total extinction during the regression, catagen phase [45]. In this context, we also have found that apoptosis signals were significantly reduced by D-panthenol treatment in cultured hair follicle cells (Figure 2A and Figure 6B). Dermal papilla is considered to resist apoptosis, associated with high levels of Bcl-2 and a lack of death receptors during the entire hair cycle in mice and humans [45,46,47,48]. However, they may be susceptible to apoptosis under certain experimental conditions [49,50] and it has been reported that minoxidil prevented cellular apoptosis in dermal papilla cells [46]. Our data suggest that the reduction of the apoptotic markers by D-panthenol treatment could explain at least in part the cellular mechanism of its clinical anti-hair loss efficacy.

D-panthenol treatment also significantly reduced the mRNA expression of cell senescence markers, p21 and p16 (Figure 2B and Figure 6C). p16 and p21 are well-known senescence markers which inhibit cyclin-dependent kinase activity. Senescence-related cell proliferation markers are decreased by the increased expression of p21 and p16 [51,52]. Indeed, balding DPCs and ORSCs, expressing high levels of p16, showed a much slower growth rate and immature hair follicle differentiation [53]. Taken together, our study suggests that D-panthenol could possibly support hair growth by stimulating the cell proliferation, suppressing the apoptosis and preventing the senescence in hair follicle DPCs and ORSCs.

Alkaline phosphatase activity is a well-known anagen marker of dermal papilla. We have found that D-panthenol treatment increased ALP activity and also up-regulated the mRNA expression of ALP (Figure 3A,B). The expression of versican, another dermal papilla anagen marker, was also elevated by D-panthenol treatment both in protein and mRNA levels (Figure 3C,D). Since Wnt/β-catenin pathway activation plays a crucial role for maintaining anagen in the dermal papilla, we investigated the effect of D-panthenol on Wnt/β-catenin signaling. Up-regulated β-catenin protein level was confirmed by both immunostaining and Western blotting (Figure 3C,D). The precise mechanisms, however, need to be elucidated. Our results demonstrate that D-panthenol could assist anagen phase establishment and maintenance by stimulating ALP, versican expression, and Wnt/β-catenin signaling.

Not only anagen maintenance, but also catagen inhibition could be a main strategy for anti-hair loss therapies. TGF-β1 expressed in bald DPC is known to stimulate catagen entry of the hair follicle. We have found that D-panthenol significantly reduced TGF-β1 expressions in both mRNA and protein levels (Figure 4A,B). Taken together, our data suggest that D-panthenol could support hair growth by inhibiting catagen entry, elongating anagen phase.

Among growth factors, VEGF is reported to stimulate cell proliferation both in the dermal papilla and outer root sheath. VEGF exerts its effects through VEGF receptor-2, a receptor tyrosine kinase expressed in both the dermal papilla and outer root sheath. It was revealed that the expression of VEGF was up-regulated by D-panthenol treatment not only in hDPCs but also in hORSCs (Figure 5 and Figure 7B). The enhanced expression of VEGF in ORSCs, even though only in mRNA levels, is not a commonly reported event whose implication should be elusive. In addition, the expression of VEGF-R2 (KDR) was also up-regulated by D-panthenol in cultured hORSCs. Our results demonstrate that D-panthenol could support hair growth by stimulating the VEGF signals, which is important to determine hair follicle size by angiogenesis in both hDPCs and hORSCs.

In the present study, we demonstrated the in vitro hair growth-supporting activities of D-panthenol and elucidated several underlying cellular and molecular mechanisms of D-panthenol. In spite of the in vitro evidence, our study has major limitations since neither in vivo mimicking systems, such as human hair follicle organ culture, nor an animal model have been adopted for further elucidation. Despite these limitations our data still have meaningful implications that our findings do provide some cellular and molecular clues of D-panthenol for its hair growth stimulating activities which have not been demonstrated yet.

**Figure 8:**
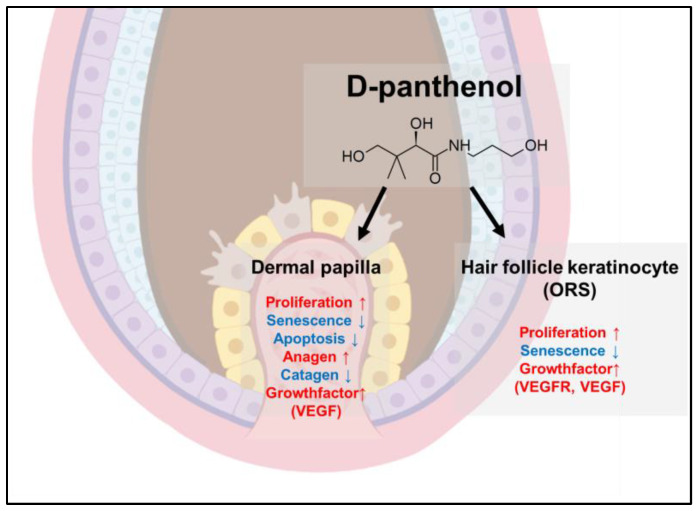
Summarized in vitro effects of D-panthenol in hair follicle cells. All these effects in combination could lead to hair growth promotion. ↑:upregulation, ↓:downregulation.

In conclusion, our findings demonstrate that D-panthenol stimulated the cell proliferation and decreased the expression of cell senescence markers such as p16 and p21 both in cultured hDPCs and hORSCs. D-panthenol reduced the expression of apoptosis and senescence markers, increasing the expression of anagen markers and decreasing the expression of catagen-inducing factors in cultured hDPCs. Furthermore, D-panthenol stimulated the expression of VEGF in both hDPCs and hORSCs, additionally up-regulating VEGF-R expression in cultured hORSCs. Our data demonstrate that D-panthenol could promote hair growth by prolonging the anagen phase and preventing catagen entry by stimulating the dermal papilla and outer root sheath cells. The effects of D-panthenol on hair follicle cells are summarized in Figure 8.

## Data Availability

The datasets used and/or analyzed during the current study are available from the corresponding author on reasonable request. Some data may not be available because of the policy of company and ethical restrictions.
